# Effect of farm size on vulnerability in beekeeping: Insights from mediterranean Spain

**DOI:** 10.1007/s13280-024-02099-0

**Published:** 2024-12-11

**Authors:** Feliu López-i-Gelats, Erik Hobbelink, Paula Llaurador, Marta G. Rivera-Ferre

**Affiliations:** 1 Agroecology and Food Systems Chair, Faculty of Health Sciences and Welfare, Universitat de Vic-Universitat Central de Catalunya, C/de la Laura 13, 08500 Vic, Spain; 2 INGENIO (CSIC-Universitat Politècnica de València), Edifici 8E, Acc. J, 4ª Planta Ciutat Politècnica de la Innovació (CPI), Camí de Vera, s/n, 46022 València, Spain; 3 Associated Unit “Innovaciones Transformativas y Comunidades Inclusivas”, Edifici 8E, Acc. J, 4ª Planta Ciutat Politècnica de la Innovació (CPI), Camí de Vera, S/N, 46022 València, Spain

**Keywords:** Adaptation, Agrocology, Climate change, Honeybees, Pollination

## Abstract

There is broad consensus on the socioeconomic importance of beekeeping and the essential services it provides through pollination. However, beekeeping is being impacted by global environmental changes, and more specific insights are needed. Beekeeping is not a homogeneous activity; it is practiced in various ways and contexts. This study examines how these changes affect beekeeping operations of different sizes in Mediterranean Spain and whether size influences adaptation. Structured interviews were conducted to assess the sector's vulnerability, with climate change identified as a key contributing factor. The findings reveal that the vulnerability of beekeeping is shaped not only by multiple transformations but also by the diverse adaptive capacities of beekeepers. Additionally, the size of beekeeping operations significantly influences this capacity, a factor that should be integrated into policymaking given the sector’s highly polarized nature.

**Supplementary Information:**

The online version contains supplementary material available at 10.1007/s13280-024-02099-0.

## Introduction

Beekeeping is an activity of great socioeconomic importance that also provides fundamental services through pollination (IPBES 2016), biodiversity conservation (Balvanera et al. 2005; Kremen 2005; Potts et al. 2016), and food security (Aizen and Harder 2009; Gallai et al. 2009; Lautenbach et al. 2012; Klatt et al. 2014; Potts et al. 2016; Feketéné Ferenczi et al. 2023). Moreover, beekeeping has a notable economic significance. In Europe, there are approximately 711 000 beekeepers, 97% of whom are not professionals, i.e., those with fewer than 150 honeybee colonies. Germany and Poland, with 149 000 and 91 000, respectively, are the countries with the most beekeepers. European honey production, which amounted to 285 500 tons in 2022 (European Commission 2023), accounts for 16% of world honey production. The importance of beekeeping has been growing both in terms of honey production—from 235 500 tons in 2014 to 285 700 tons in 2022—and in the number of beekeepers—from 635 638 in 2010 to 710 825 in 2022 (European Commission 2023). Spain, with 28 786 beekeepers and almost 3 million honeybee colonies in 2018, has the most colonies out of any country in Europe and the highest degree of beekeeping professionalization. While the average number of honeybee colonies per professional beekeeper is 386, in the case of nonprofessional beekeepers, it is 33. The percentage of professional beekeepers was 23% (2018), and the number of both professional and nonprofessional beekeepers has been increasing in recent years. In particular, beekeeping in Mediterranean Spain comprises one-third of Spanish beekeepers, 42% of honeybee colonies, and half of the honey production of the country (MAPA 2021). However, notably different management strategies exist: beekeeping is a heterogeneous activity that is very often linked to the size of the operation, i.e., the number of colonies managed. In this context, there are more extensive management systems, usually smaller, with fewer honeybee colonies and a strong focus on honeybee welfare. On the other hand, there are more intensive management systems that involve managing a larger number of honeybee colonies that are also more populated.

Beekeeping provides fundamental ecosystem services due to role of honeybees as pollinators (Balvanera et al. 2005; Kremen 2005; Potts et al. 2016; Hung et al. 2018; Feketéné Ferenczi et al. 2023). For instance, pollination is fundamental for food security. It is estimated that 60–90% of wild plants (Kearns et al. 1998; Potts et al. 2016) and 70% of the world's most common crops, which account for 35% of food production and including vegetables, fruits, and crops such as rice, wheat, corn, or potatoes, depend on pollination (Ashman et al. 2004; Klein et al. 2007). Feurbacher et al. (2024) estimated a reduction in global average crop yields of 9%, which would lead to a 6% expansion in cropland due to a worldwide decline in both managed and wild pollinators by 2030. It is estimated that 71 out of the 100 crop species that provide 90% of the world’s food depend on honeybee pollination (Klein et al. 2007). Several studies have attempted to calculate the economic value of pollination as a global ecological service. Constanza et al. (1997) were among the first to do so, estimating the value of 88 000 million euros. Later, Gallai et al. (2009) increased this figure to 115 000 million, which is equivalent to 9.5% of the value of global food production. Lautenbach et al. (2012) estimated the value of pollination at 265 000 million euros, and Feurbacher et al. (2024) estimated that a decrease in pollinators would reduce global economic welfare by 302 billion EUR (0.4% of the global GDP). Additionally, the volume of agricultural production dependent on animal pollination has increased by 300% in the past 50 years, yet pollinator-dependent crops show lower growth and yield stability than those that do not rely on pollinators (Potts et al. 2016). Specifically, in Europe, the economic contribution of honeybees to agriculture through pollination was estimated at 22 000 million euros (European Commission 2013). In Spain, this contribution varies between 4 000 and 24 000 euros per square kilometer of cultivated land (Leonhardt et al. 2013).

Although the number of honeybee colonies has increased by approximately 85% in the last 60 years worldwide (Phiri et al. 2022), rising mortality has also been reported (Chauzat et al. 2013). Sudden losses of large numbers of honeybees in the early 2000s, primarily in the United States and Europe (Hendrikx et al. 2009; Pizarro and Montenegro 2012), increased awareness of the vulnerability of honeybees and beekeeping to a variety of climate and non-climate stressors. Indeed, changes in climate must be considered alongside other social, political, and ecological transformations, which are occurring in parallel and jointly shape the landscape where honeybees are kept and beekeeping is practiced, portraying vulnerability as multidimensional (UNDRR 2021; Quandt and Paderes 2023). This notion of contextual vulnerability (IPCC 2014) proposes an integrated approach to interpret the links between nature and society, i.e., as a feature of complex socioecological systems, which are increasingly used to holistically understand the implications of global environmental change in specific domains (López-i-Gelats et al. 2016; Pandey et al. 2016; Rufat et al. 2019; Birkmann et al. 2022). In this context, the literature points to diverse global transformations that are driving honeybee decline (Potts et al. 2010; González-Varo et al. 2013; Henry et al. 2012): (i) climate change (Memmott et al. 2007; Hegland et al. 2009; Van Espen et al. 2023), mainly through the decoupling of honeybee phenology and flowering periods (Fitter and Fitter 2002; Menzel et al. 2006); spatial mismatches between honeybees and pollinated species (Fitter and Fitter 2002; OECC 2008); changes in the distribution and virulence of pathogenic species such as *Nosema cerana* (Martín-Hernández et al. 2009); reduced survival of non-native invasive species (Walther et al. 2009); or food shortages (Rami Reddy et al. 2012; Gómez et al. 2013); (ii) the relationship between the increase in atmospheric CO_2_ and a reduction in the protein content of pollen should also be considered (Ziska et al. 2016); and (iii) the spread of diseases (Cameron et al. 2011), with varroosis, virosis, and nosemosis being the most commonly reported (Higes et al. 2010; O’Shea-Wheller et al. 2022); (iv) the spread of invasive non-native species (Moron et al. 2009), including mammals (e.g., *Meles meles* or *Ursus arctos*), insectivorous birds (e.g., *Merops apiaster*), lepidoptera (e.g., *Acherontia atropos* or *Galleria mellonella*), different types of wasps (e.g., the Asian wasp *Vespa velutina*) or coleoptera (e.g., the *Cetonia melicivorus* beetle); (v) habitat loss and fragmentation (Garibaldi et al. 2011), which result in reduced availability (both in quantity and quality) of pollen and nectar for honeybees; and (vi) intoxication, primarily due to industrial agriculture (Kremen et al. 2002; Tscharntke et al. 2005; Gill et al. 2012; Whitehorn et al. 2012; Goulson et al. 2018). Honeybees also suffer from Colony Collapse Disorder (Hendrikx et al. 2009; UNEP 2010; Pizarro and Montenegro 2012). Finally, the fact that beekeepers tend to generate situations of honeybee overpopulation makes beekeeping an additional stressor for wild bees and their capacity for pollination, possibly reducing both wild bee diversity and population (Russo 2016; Geslin et al. 2017; Alaux et al. 2019). In summary, the capacity of honeybees to perform their relevant contributions in terms of economic return, cultural identity, and ecosystem services is largely dependent on a set of ongoing changes and trends.

In the European context, previous studies have analyzed the vulnerability of beekeeping to climate change (Van Espen et al. 2023), and the relatively high vulnerability of the Mediterranean region is widely recognized, with climatic conditions often exceeding the optimal temperature for nectar secretion in many floral species (Flores et al. 2019; Gérard et al. 2020; Novelli et al. 2021). Other drivers of change, such as culture, education, or gender (Mburu et al. 2017; Olana and Demrew 2018; Gross 2020), have also been analyzed. Guiné et al. (2021) conducted a detailed characterization of beekeeping and associated activities in Europe. However, there remains a lack of knowledge on whether and how different farming systems, defined by the size of the operation, experience varying vulnerabilities to both climate and non-climate drivers. Indeed, Velardi et al. (2021) showed that management decisions in beekeeping are related to the size and scope of beekeepers’ operations, as well as to the ways in which they value their bees. Thus, we hypothesized that size also affects decisions regarding the transformations associated with global environmental change. Considering all the above, the objective of this work is to characterize the vulnerability of beekeeping to global environmental change in Mediterranean Spain and evaluate whether and how the size of operations (which are used as a proxy of the intensification degree of the production system) may influence vulnerability and the adaptation strategies implemented by beekeepers. In doing so, we aim to test the hypothesis that different beekeeping production systems suffer varying impacts from these transformations; thus, adaptation strategies are also diverse. In particular, we compared large (> 150 colonies), medium (25–150 colonies), and small (< 25 colonies) beekeepers in Mediterranean Spain.

## Materials and methods

**Fig. 1:**
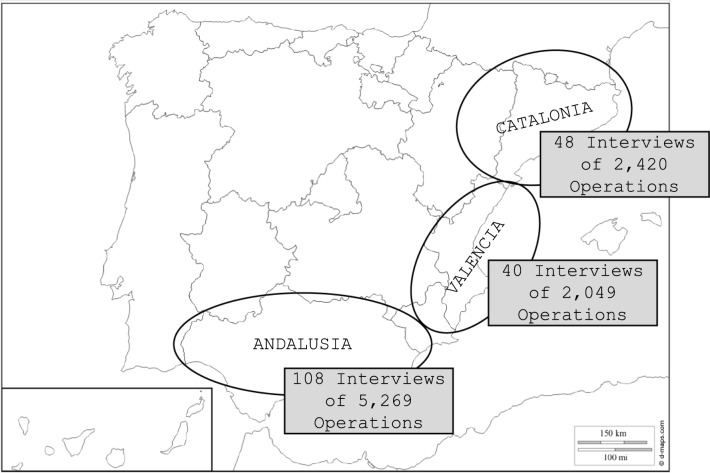
Location of the sample of beekeepers interviewed in Mediterranean Spain *Note:* According to data from MAPA (2021)

To examine the effect of farm size on the vulnerability of beekeeping in Mediterranean Spain to global environmental change, structured interviews were conducted. A total of 196 structured interviews were conducted with beekeepers from Mediterranean Spain, particularly from the regions of Andalusia, Valencia, and Catalonia, between 2016 and 2020 (Fig. 1). The structured interviews were conducted in person, taking advantage of various meetings of beekeeping associations. The interviews were not recorded, as it was recognized that beekeepers felt more comfortable with this medium. This approach was possible, as the structured interview consisted of yes/no questions (see the Supplementary Information). However, the interviewer took notes of the answers to the questionnaire, and an assistant was always present. The interviews were conducted in the native languages of the interviewees—Spanish or Catalan, depending on the region—and lasted approximately half an hour. The number of interviews conducted in each region was established based on the number of beekeepers in each region. Given that the entire study area comprises a total of 9758 operations (MAPA 2021), with Andalusia, Valencia, and Catalonia accounting for 54%, 21%, and 25%, respectively, the same percentages were employed to set up the sampling effort. Thus, 108 interviews were conducted in Andalusia, 40 in Valencia, and 48 in Catalonia.

To design the interviews, two focus groups with key informants (three major beekeeping experts—one from each of the three regions under consideration—and members of the Spanish Office for Climate Change) were held in Valencia and Madrid in June 2015 and October 2016 to agree on the specific items to be included in the interviews. The interviews were designed to collect data on four main domains, reflecting an approach to the beekeeping sector as a complex socioecological system: (i) a general description of the key attributes of beekeeping operations, including aspects such as workforce availability, number of honeybee colonies, type of production, commercialization channels used, and attitudes toward their activity; (ii) exposure, with the characterization of the fundamental changes impacting both beekeeping, including climate, policy, economy, social, ecology, and management elements; (iii) adaptation, which entailed listing the practices that the beekeepers implement to cope with or benefit from the transformations; and (iv) sensitivity, which involves identifying the main obstacles to and potential opportunities in beekeeping, as well as the needs and possibilities identified by beekeepers in the sector. A total of 151 categorical binary variables emerged from the yes/no questions. Only variables with a frequency greater than 5% were considered explanatory, and 137 categorical binary variables were retained for the analysis. With the SPAD 5.5 software package, descriptive statistics were obtained to characterize the vulnerability of honeybee operations in the sample. A chi-square statistical analysis comparing groups of operations of different sizes was subsequently performed to capture whether and how the size of the operation affected vulnerability and adaptation options. Three groups of honeybee operations were considered: those with fewer than 25 colonies, those with between 25 and 150 colonies, and professional beekeepers with more than 150 colonies.

## Results

### General description

Among the beekeepers interviewed, 97% produce honey, and 61.7% also produce other types of products or services, mostly pollen, propolis, pollination for agriculture, royal jelly, and swarms. Pollen and swarm production are the main alternatives. Self-consumption and direct sales are the most common forms of commercialization, with 61% and 64%, respectively. Only beekeepers running professional honeybee operations dedicate their efforts full-time to beekeeping. All other beekeepers combine beekeeping with other economic activities. Moreover, professional honeybee operations tend to have a stronger family tradition. Another relevant aspect is that 17% of beekeepers cite non-economic reasons as their primary motivation for engaging in this activity, specifically, the existence of a long family tradition, beekeeping as a valuable hobby, and beekeeping as a lifestyle that allows them to be closer to nature. Generally, the number of honeybee colonies per beekeeper in Mediterranean Spain has been slightly increasing (Table 1).

**Table 1:** Main features of honeybee operations in the Spanish Mediterranean according to the number of honeybee colonies managed

	< 25 (24.5%)	25 ≤ and ≤ 150 (17.3%)	> 150 (58.2%)
*Workforce *			
Family tradition	14.6***	38.2	75.4***
Full-time commitment	0.0***	14.7**	50.9***
> 1 worker	29.2	35.3	41.2
> 2 workers	8.3	8.8	16.7
With salaried workers	8.3	2.9	21.9***
*Trends in no of colonies *			
Increasing number of colonies	22.9	23.5	14.0
Steady number of colonies	37.5	41.2	54.4
*Production *			
Honey	97.9	97.1	96.5
Pollen	14.6	11.8	25.4
Propolis	33.3***	8.8	14.0
Pollination	4.2	0.0	14.0**
Royal jelly	2.1	0.0	7.9
Wax	20.8**	35.3	44.7**
Beekeeping services	2.1	5.9	0.0
Swarms	0.0	0.0	13.2***
*Commercialization *			
Direct selling	37.5***	85.3**	68.4
Through intermediary	12.5***	11.8***	54.4***
Self-consumption	97.9***	79.4**	39.5***
Through cooperative	0.0	0.0	8.8**
*Vocation*			
In touch with nature	14.6	14.7	12.3
Family tradition	8.3**	29.4	32.5**
Hobby	79.2**	73.5	52.6***
Working option	12.5	11.8	21.1
Generational succession	87.5***	57.6	57.1**

*p < 0.05, Chi-square test. **p < 0.01, Chi-square test. ***p < 0.001, Chi-square test

### Exposure to climate and non-climate stressors

**Fig. 2:**
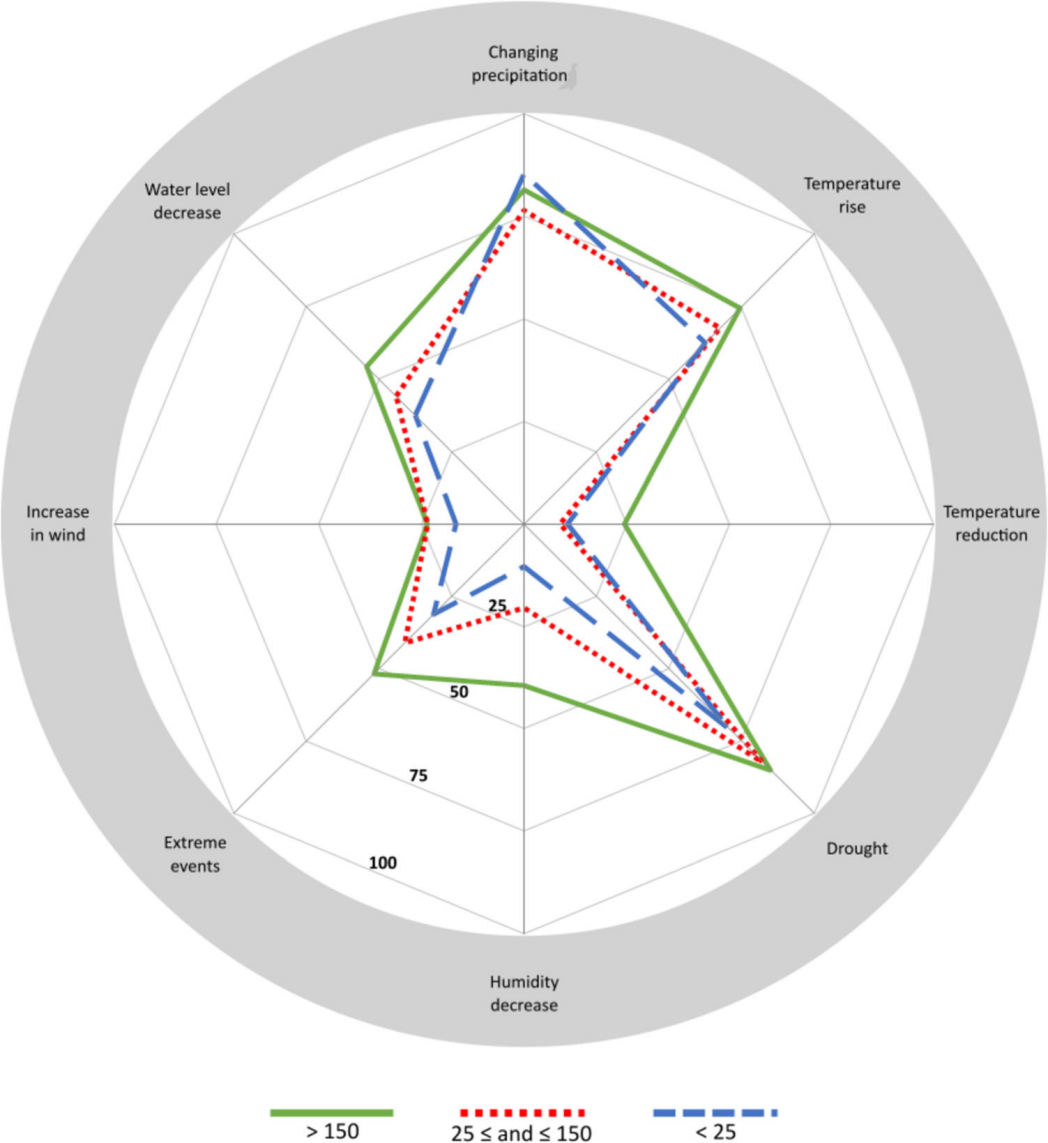
Climate trends affecting the Spanish Mediterranean beekeeping sector according to the opinion of beekeepers managing different-sized honeybee operations *Note*: See Table 2 for a comprehensive table

A total of 51 different transformations were reported by at least 5% of the beekeepers interviewed (Table 2). Most of the beekeepers highlighted the displacement of seasons, especially the irregularity of precipitation (82%), rising temperatures (70%), and drought (81%), as climate trends are increasingly affecting beekeeping (Fig. 2). There is much less consensus concerning other climate trends. See Table 3 for the main expected (according to the literature) and observed (according to the interviewed beekeepers) climate trends and their impacts on beekeeping.

**Table 2:** Main transformations undergoing the beekeeping sector in the Spanish Mediterranean according to the opinion of beekeepers managing different-sized honeybee operations

	< 25 (24.5%)	25 ≤ and ≤ 150 (17.3%)	> 150 (58.2%)
*Climate*			
Changes in seasonality of precipitations	85.4	76.5	81.5
Rise in temperature	62.5	67.6	74.5**
Drought	70.8	82.3	85.0
Water level decrease	37.5	44.1	54.3
Extreme events	31.2	41.1	51.7
Reduction in temperature	10.6	8.8	24.5
Humidity decrease	10.4**	20.5	39.4***
Increase in wind intensity	16.6	23.5	23.6
Greater humidity	0.0	0.0	5.0
*Policy*			
Inappropriate legislation	62.5	80.0	70.0
Marginalization	50.0	50.0	46.4
Outdated sanitary programs	54.1	47.0	49.1
Agro-environmental schemes	10.4***	26.4	58.7***
Organic production	25.0	38.2	32.4
Subsidy hunting	10.4**	23.5	28.9
Hyper-sanitary regulations	6.3**	23.5	28.9
Pollination premium	6.2***	14.7	35.9
Local product labels	20.8**	38.2	42.1
Bee-eater coexistence regulation	8.5	20.5	21.9
*Economy*			
Pesticides	93.0***	82.4	93.0***
Monocultures	50.0	50.0	52.6
Uninformative labels	58.3**	64.7	80.7***
Poor quality honey commercialization	62.5***	100.0	80.0***
Settlement conflicts	29.2**	41.2	50.9
Overexploitation	31.3	44.1	45.6
GMOs	35.4	35.3	54.4**
Honey adulteration	54.2***	82.4	86.8***
Theft	31.3**	38.2	58.8***
Shift to crops less adequate for bees	31.3	44.1	46.5
Fuel price rise	16.7***	50.0	64.0***
*Social*			
Rising demand for local products	72.6	64.7	58.8
Sensitivity of beekeepers in organic production	43.8	35.3	45.6
People’s awareness of honeybees	62.5	73.5	72.8
Rising demand for healthy products	58.3	61.8	64.9
Rising demand for organic products	54.2	52.9	45.6
Increase in training	60.4**	47.1	33.3**
Generational succession with limited experience	18.8	11.8	23.7
*Ecology*			
Spread of diseases	79.2	79.4	81.6
Concentration of the flowering period	39.6	44.1	46.5
Spread of species hostile to the honeybees	79.2	70.6	71.9
Colony collapse	66.7	70.6	78.1
Reduction of flowering length	62.5**	76.5	81.6
Disappearance of wild colonies	62.5	79.4	59.6
Loss of habitats	58.3	64.7	58.3
Hybridization	20.8	14.7	28.9
Pollination crisis	18.8**	26.5	40.4**
Spread of exotic species	58.3**	50.0	30.7***
Wildfires	18.8	23.5	36.0
*Management*			
Inappropriate application of sanitary treatments	62.5	67.6	71.9
Inappropriate honeybee welfare management	56.3	55.9	46.5
Employment of foreign breeds of honeybees	31.3	17.6	16.7
Increase of prophylaxis in treatments	29.2	47.1	43.0

^*^p < 0.05, Chi-square test. **p < 0.01, Chi-square test. ***p < 0.001, Chi-square test

**Table 3:**
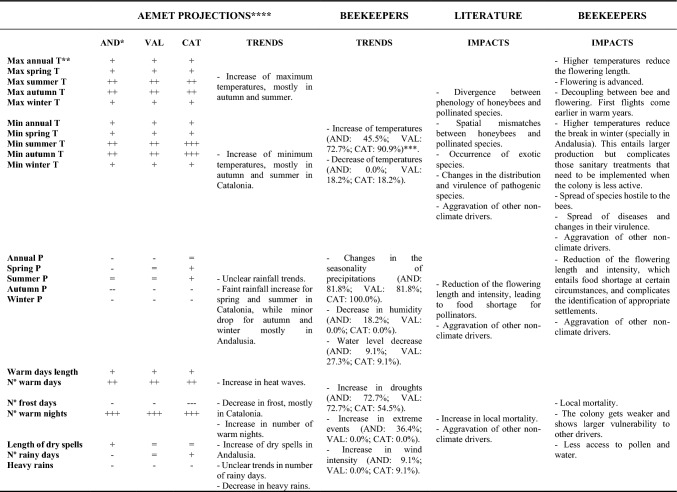
Main expected and observed climate trends and their impacts on beekeeping *Note* = means no change; + means increasing intensity; and –means decreasing intensity. *AND means Andalusia; VAL means Valencia; and CAT means Catalonia. ** Max annual T is Maximum annual temperature; Min annual T is Minimum annual temperature; P means precipitations. *** Percentage of people interviewed identifying the given climate trend as relevant for the beekeeping activity per each region in brackets. ****AEMET: Meteorology Spanish Agency (2023)

Non-climate stressors refer to changes occurring in the policy, economic, social, and ecological domains, as well as changes in the management of honeybee operations. In the policy domain, professional beekeepers (> 150 colonies) mention marginalization, i.e., regulations not adapted to the reality of beekeeping, identifying the pollination premium and agri-environmental measures as policy-related issues most affecting their activity. However, medium-sized operations identify the existence of subsidy hunters, poorly adapted regulations, outdated sanitary programs, and hyper-sanitary regulations.

**Fig. 3:**
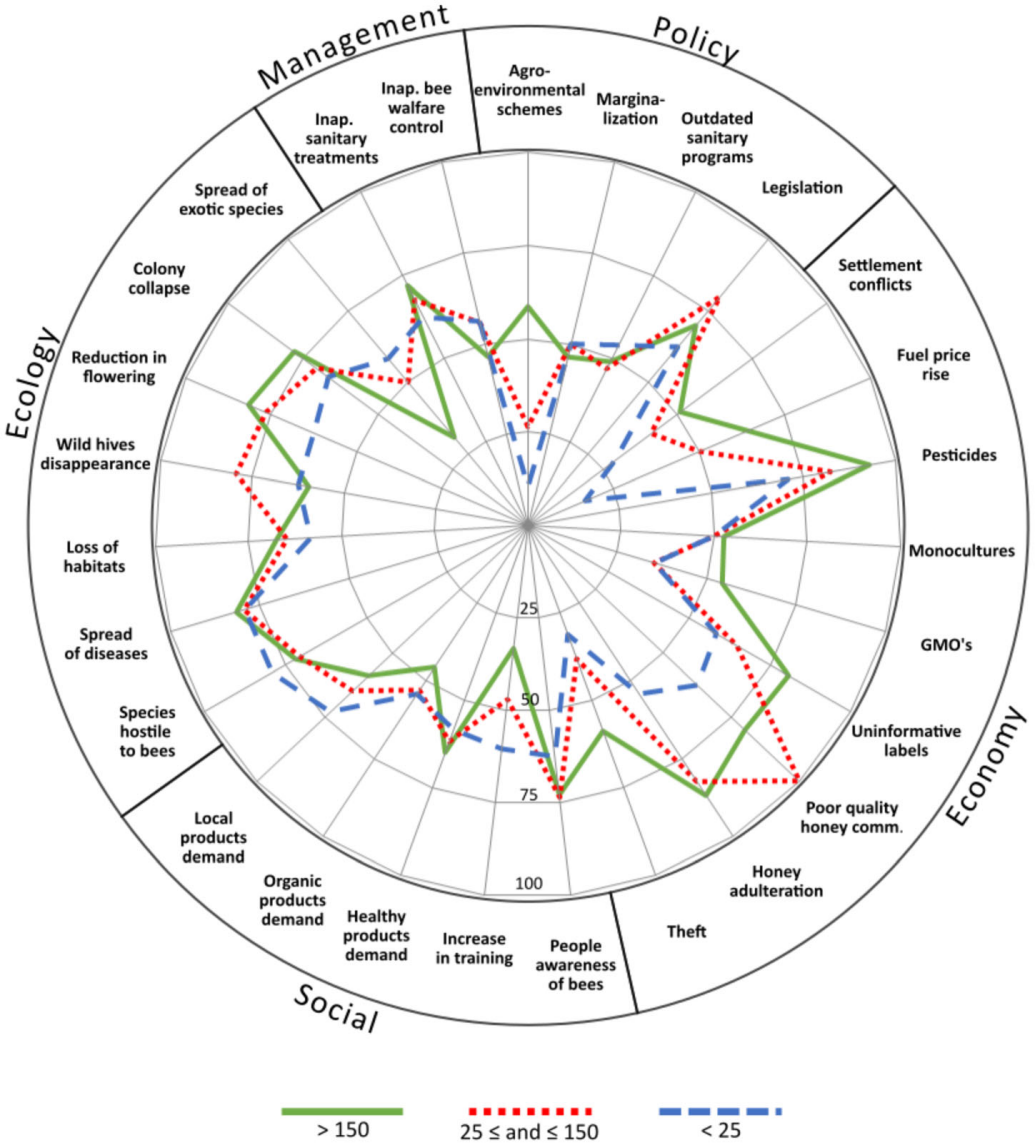
Main non-climate transformations affecting the Spanish Mediterranean beekeeping sector according to the opinion of beekeepers managing different-sized honeybee operations *Note*: Only those transformations being mentioned at least by 75% of the beekeepers of at least one of the honeybee operation types considered appear in this figure (see Table 2 for a comprehensive table)

In the economic domain, the interviewees identified many transformations: coexistence with industrial farming, settlement conflicts, competition with low-quality and very cheap imported honey, and theft of honeybees and honeybee colonies (Fig. 3). Virtually 100% of the interviewees stated that it is impossible to coexist harmoniously with industrial farming, most notably due to the use of insecticides. Beekeepers frequently mentioned crops such as rapeseed, corn, and cotton as having harmful consequences for neighboring apiaries.

In the social domain, both medium-sized (25–50 colonies) and smaller honeybee operations (< 25 colonies) are perceived as being the most exposed to these types of stressors (Fig. 3). Increased awareness of the importance of honeybees and other pollinators, growing interest in local and organic products, increased sensitivity to organic beekeeping, and increased demand for healthy products are the primary changes mentioned.

With respect to the ecological drivers, while there is a consensus that the spread of diseases is a very relevant driver, professional beekeepers place more importance on the spread of enemy species, such as bee-eaters (*Merops apiaster*). Beekeepers with fewer than 25 honeybee colonies emphasize the effects of the shortening and concentration of the flowering period on their activity, whereas medium-sized operations emphasize the effects of habitat loss and fragmentation, the disappearance of wild honeybees, the pollination crisis (including wild pollinator insects), the hybridization of the black bee (*Apis mellifera mellifera*) with other improved breeds (seen as making it lose some of its traditional qualities), and the spread of exotic species (such as the Asian wasp *Vespa velutina*).

With respect to colony management, beekeepers of all three types of operations agreed that some of their colleagues are not rigorous in their sanitary efforts and make inadequate use of sanitary treatments, which is seen as a potential source of disease spread. Small honeybee operations also point to the use of foreign breeds of honeybees as a very important transformation. Finally, it is important to note the difference between professional and non-professional beekeepers in terms of the increase in prophylaxis in treatments. While this is practically insignificant for professionals, the rest of the beekeepers consider it a relevant transformation. Prophylaxis is carried out by managing density at four levels: colony, apiary, settlement, and during mobility.

### Adaptation strategies

**Fig. 4:**
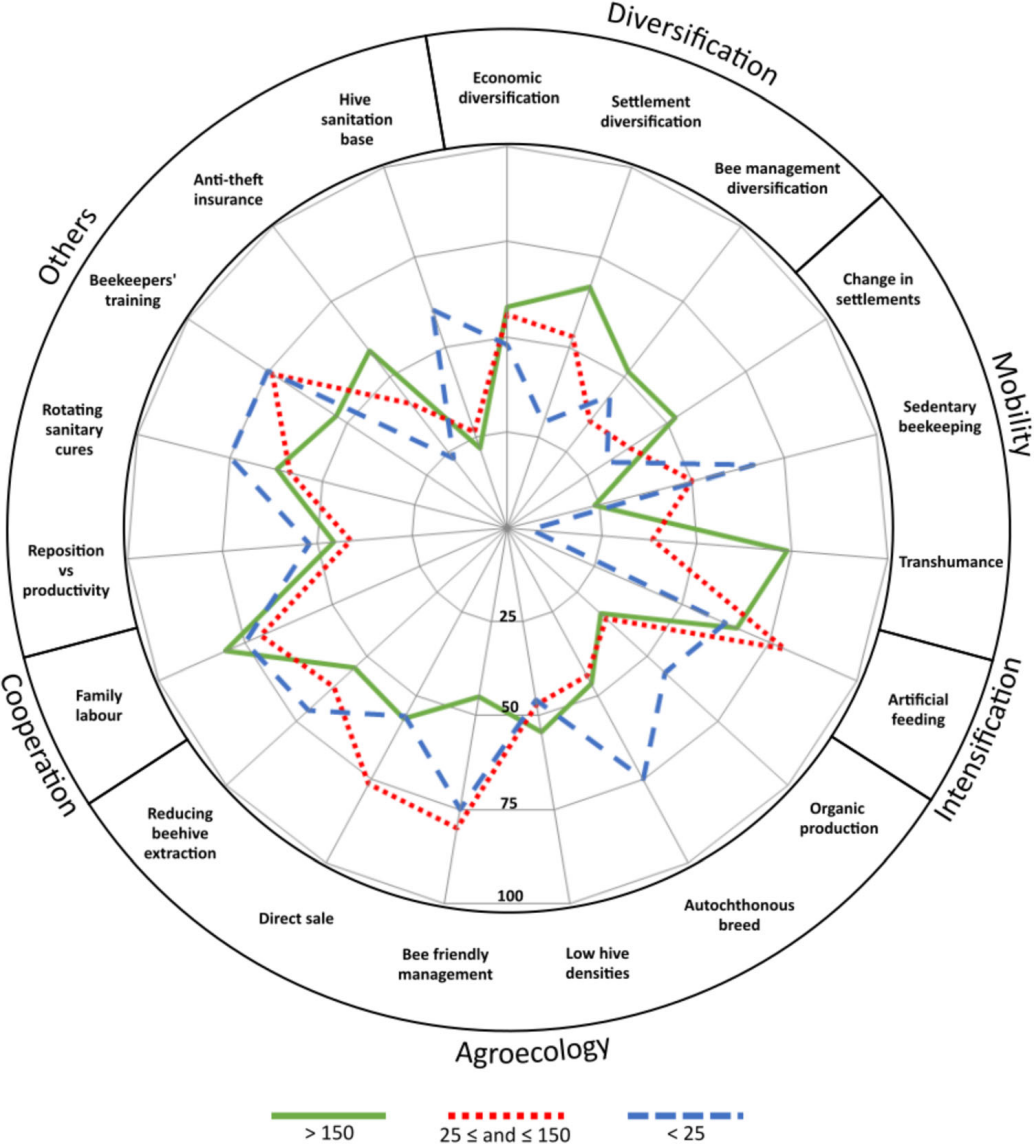
Main adaptation strategies identified in the Spanish Mediterranean beekeeping sector according to the opinion of beekeepers managing different-sized honeybee operations *Note*: Only those adaptation strategies being mentioned at least by 75% of the beekeepers of at least one of the honeybee operation types considered appear in this figure (see Table 4 for a comprehensive table)

A total of 43 different adaptation strategies were reported by at least 5% of the interviewed beekeepers in Mediterranean Spain as being employed to address ongoing transformations (Table 4). These strategies can be divided into five main groups (Fig. 4): (i) diversification; (ii) mobility; (iii) intensification; (iv) agroecology; and (v) cooperation.

**Table 4:** Adaptation strategies implemented by the beekeeping sector in the Spanish Mediterranean according to the opinion of beekeepers managing different-sized honeybee operations

	< 25 (24.5%)	25 ≤ and ≤ 150 (17.3%)	> 150 (58.2%)
*Diversification *			
Economic diversification	47.9	55.9	57.9
Beehive product diversification	37.5	23.5	44.7
Settlement diversification	29.2***	52.9	66.7***
Specializationin unconventional beehive products	18.8	17.6	21.9
Honeybee management diversification	43.8	35.3	51.8
Diversification in manufactured products	25.0	17.6	24.6
*Mobility *			
Transhumance	6.3***	38.2	73.7***
Change in settlements	31.3	38.2	52.6**
Sedentary beekeeping	66.7***	50.0	23.7***
Change in transhumance routes	4.2***	20.6	40.4***
Mobility increase	12.5**	32.4	37.7**
*Intensification *			
Artificial feeding	62.5	79.4	65.8
Greater use of inputs	22.9	29.4	28.9
Purchase of new lots of honeybees	20.8	23.5	12.3
Wholesaling	0.0**	8.8	21.1***
Purchase of foreign breeds of honeybees	4.2	5.9	6.1
*Agroecology*			
Autochthonous breed	75.0***	44.1	46.5
Direct sale	56.3	76.5	57.0
Managing according to bee’s nature	75.0**	80.0	45.0
Colony location	47.9	47.1	41.2
Low colony densities	45.8	47.1	54.4
Consumer sensitization	41.7	32.3	33.3
Animal welfare	39.6	29.4	37.7
Organic production	56.3**	35.3	33.3
Avoiding certain settlements	31.3	29.4	35.1
Low apiary densities	35.4	35.3	45.6
High-quality brands	27.1	29.4	31.6
Reducing beehive extraction	70.8	61.8	54.4
Local brand products	31.3	35.3	29.8
*Cooperation *			
Family labor	75.0	70.6	80.7
Collaboration	41.7	29.4	29.8
Bartering	22.9	44.1	32.5
Participatory management of honeybee welfare	31.3	38.2	31.6
Labor exchange	18.8	29.4	25.4
*Others *			
Beekeepers’ training	75.0	73.5	53.5**
Changing genetic line	14.6**	29.4	35.1
Rotating sanitary treatments	75.0	58.0	62.3
Anti-theft insurance	22.9***	41.2	58.8***
Hive sanitation base	60.4***	26.5	21.9***
Reposition vs productivity	52.1	41.2	45.6
Research	29.2	35.3	42.1
Colonies as signs	6.3	11.8	21.9**
Colonies to be saved	8.3	5.9	18.4

*p < 0.05, Chi-square test. **p < 0.01, Chi-square test. ***p < 0.001, Chi-square test

Diversification strategies are used by both larger and smaller honeybee operations. Although the pattern is similar in all three groups of operations, those with fewer than 25 colonies use these strategies to a lesser extent. In medium-sized honeybee operations, economic and settlement diversification is described as widespread. Professional operations notably employ the strategy of diversifying hive products. Smaller operations tend to practice more sedentary beekeeping, and as the number of colonies increases, beekeepers gradually rely more on transhumance. The use of intensification strategies, including greater use of inputs, aims to insulate production from the uncertainty of changing environmental conditions. Specifically, the most common adaptation strategies within this category include artificial feeding, increased use of inputs, and the purchase of new colonies (Fig. 4).

Agroecological strategies include practices that focus on ensuring the reproduction of honeybee colonies rather than maximizing the extraction of honey or other hive products (IPEs-Food 2016). Small and medium-sized honeybee operations adopt these strategies, especially organic production strategies, maintaining low colony and apiary densities, adapting management to the honeybee’s natural behaviors (e.g., using vertical hives), and selling high-quality brand products (Fig. 4), more often than professional operations do. Professional honeybee operations match the other two types in just two areas: the use of native honeybee breeds (such as the black bee *Apis mellifera mellifera*) and direct sales.

Cooperation strategies emphasize mutual support and collaboration among beekeepers, particularly in small and medium honeybee operations. While professional operations also demonstrate family labor dependency and, to a lesser extent, payment in kind (e.g., honey for housing colonies), operations with fewer than 25 honeybee colonies are notably involved in cooperatives and participatory health projects. Among medium-sized operations, the strategies most highlighted include participation in cooperatives, payments in kind, labor exchanges with colleagues, and collaborations on health issues (e.g., collective purchase of treatments).

### Perception of the problems, needs and future options for beekeeping

As we have described, beekeeping in Mediterranean Spain takes place in an environment where multiple transformations interact, often with unknown or uncertain implications. These transformations do not have the same consequences across all honeybee operations. Below, we examine the main problems, needs, and future options identified by beekeepers to manage different numbers of honeybee colonies (Table 5) in relation to the climatic and non-climatic transformations previously identified.

**Table 5:** Problems, needs and possibilities of the beekeeping sector in the Spanish Mediterranean according to the opinion of beekeepers managing different-sized honeybee operations

	< 25 (24.5%)	25 ≤ and ≤ 150 (17.3%)	> 150 (58.2%)
*Problems *			
Diseases	25.0	26.5	29.8
Inappropriate policies	8.3	8.8	14.9
Pesticides and industrial agriculture	12.5	5.9	11.4
Climate change	6.3	0.0	9.6
Misinformation of consumers	4.2	5.9	2.6
Inappropriate honeybee management	4.2	5.9	4.2
Exotic and invasive species	12.5**	5.9	1.8**
Origin labelling	12.5	23.5	16.7
Economic viability	6.3	8.8	9.6
*Needs *			
Valorization of honeybees	12.5	8.8	10.5
Appropriate sanitary treatments	6.3	14.7	16.7
Consumer sensitization	4.2	8.8	7.9
Reduction in bureaucracy	4.2	8.8	1.8
Beekeepers’ training	14.6	14.7	4.4**
Direct sale	14.6	0.0	7.0
*Possibilities*			
Valorization of pollination services	18.8	20.6	17.5
Highquality product with increasing demand	20.8	23.5	12.3
Organic, animal-welfare sound management	12.5**	0.0	4.4

*p < 0.05, Chi-square test. **p < 0.01, Chi-square test. ***p < 0.001, Chi-square test

**Fig. 5:**
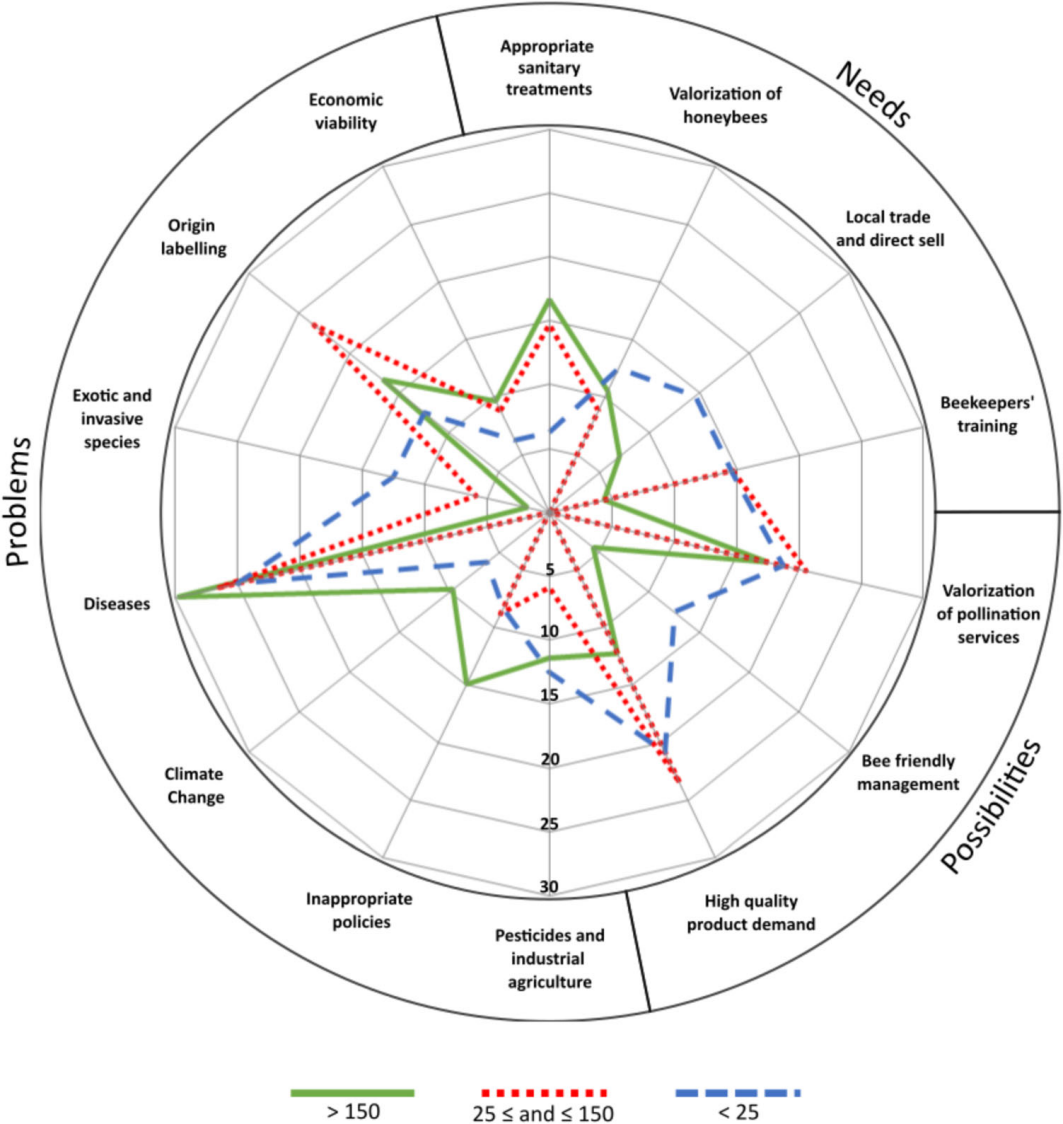
Problems, needs and possibilities of the Spanish Mediterranean beekeeping sector according to the opinion of beekeepers managing different-sized honeybee operations *Note:* See Table 5 for a comprehensive table

Beekeepers of all three operation sizes identified different problems in the sector (Fig. 5). Nevertheless, all agreed on attributing a fundamental role to the issue of coexistence with industrial farming, particularly the use of neonicotinoid insecticides, and to the emergence of diseases, many of which have only appeared recently. The emergence of inadequate policies that do not consider the nature of beekeeping is especially alarming for beekeepers managing medium-sized and large honeybee operations. Sedentary beekeepers who are not reliant on subsidies prioritize other problems. Twenty-five percent of beekeepers, mainly from large honeybee operations, pointed to climate change as a relevant challenge, especially due to its damaging effects on the quality and quantity of the honeybee diet and the increased difficulty in finding suitable settlements. Similarly, settlement conflicts are referred to as problems primarily by larger operation beekeepers, particularly those conducting transhumance. With respect to the sector’s expressed needs, medium-sized and small operations agreed on reducing bureaucracy barriers and providing more training for beekeepers. Professional beekeepers, on the other hand, primarily called for greater awareness of the role played by honeybees and pollination, as well as the health and biodiversity benefits of many hive products and services. Moreover, beekeepers highlighted the need for effective honeybee health treatments.

Despite the significant magnitude and diversity of the challenges that beekeeping faces, beekeepers have also identified several potential options for supporting this activity. Medium-sized beekeepers emphasized the need for the valuation of ecosystem services provided by honeybees, either to improve the sector’s image with consumers and policy-makers or as an economic strategy involving payment for environmental services. Small beekeepers pointed to the potential of agroecological management, emphasizing their ability to provide products increasingly demanded by consumers, as well as to offer management options based on prophylaxis—rather than the currently predominant therapeutic approach—to address the multiple current threats.

## Discussion

Our results show that the size of the beekeeping operation, which is used as a proxy of the degree of intensification, has a greater influence on the adaptive capacity of beekeepers than on their perception of the main transformations impacting the sector. The group of transformations (both climate and non-climate) undermining beekeeping activities, as identified by beekeepers, generally aligns with those highlighted in the literature (IPBES 2016). Concerning the transformations linked to climate change, it is interesting to emphasize the large degree of consensus between the scientific literature and the beekeepers’ responses. The changing precipitation patterns, drought, and temperature rise, with the subsequent displacement of seasons, were the climate trends that were emphasized more during the interviews (Fig. 2), and they are also those most anticipated by scientific projections (AEMET, 2023). This correspondence is not surprising, given that beekeepers’ local and situated knowledge, along with their close connection to the surrounding environment, have been widely recognized (Park and Yeo-Chang 2012; Lehébel-Péron et al. 2016; Maderson 2023a). Large beekeepers tend to be more aware of the implications of climate drivers for beekeeping, probably because of their greater land and food requirements and because their earnings directly depend on these changes. There is also a broad consensus on the implications of climate transformations for beekeeping. Both beekeepers and the literature (e.g., Menzel et al. 2006; Le Conte and Navajas 2008; Martín-Hernández et al. 2009; Walther et al. 2009; Gómez et al. 2013; Goulson et al. 2015; Hevia et al. 2016; Bezner-Kerr et al. 2022) emphasize the following implications: (i) an increasing gap between honeybee phenology and pollinated species; (ii) changes in the distribution and virulence of pathogenic species; (iii) the spread of species hostile to honeybees; and (iv) food shortages at certain times of the year. However, climate change was not mentioned by the beekeepers as the most pressing transformation they are facing (Table 2).

Our study highlights the existence of a significant gap in the literature regarding the transformations occurring within the socioeconomic and policy domains that impact beekeeping (Fig. 3). The damaging consequences of inappropriate policies, or those not tailored to the needs of beekeeping, were widely reported during the interviews, but this issue is poorly addressed in the literature. To bridge the gap between beekeeping and agricultural policies, beekeepers could participate in co-producing such policies. However, various barriers, including epistemological differences, policy structures and processes, and wider systemic barriers, may hinder their participation, as reported in the UK (Maderson 2023b). Beekeepers also highlighted the need to be aware of several societal trends, such as increasing demand for local, organic, and healthier products, as well as the growing social awareness of the key role that honeybees play as pollinators. Importantly, these trends may vary across countries and even regions, as noted by Perichon et al. (2024), who identified significant differences between Northern and Southern European beekeeping countries, with greater dissatisfaction with public policies in the latter. Integrating beekeepers’ environmental values into policy-making could present an opportunity not only to support the sector but also to promote sustainable land management (Maderson 2023a) and align with EU strategies such as the “Biodiversity Strategy.”

From an economic perspective, the challenge of coexistence between industrial farming and beekeeping is well documented in the literature (e.g., Whitehorn et al. 2012; Goulson et al. 2018) and was highlighted by all beekeepers in our study. However, other transformations reported by beekeepers, such as the rise in fuel prices and the prevalence of low-quality honey with misleading labels in the marketplace, tend to be dismissed in the literature. The similarities among the three groups of beekeepers concerning the main non-climate transformation affecting beekeeping are substantial (Fig. 3). Notable differences in perception relate to agro-environmental schemes, which only professional beekeepers can access, as well as settlement conflicts and colony theft, which primarily affect transhumant beekeepers. Medium-sized beekeepers, who are often focused on quality, are more likely to repot issues with low-quality honey in the marketplace. Notably, many of the subsidies designed for the sector can only be requested by operations with more than 150 colonies, meaning that they are largely inaccessible to nonprofessional beekeepers. However, certain grants, such as agro-environmental and pollination premiums, along with regulations concerning local products, are highly valued by beekeepers. This suggests the coexistence of diverse discourses on farming policy, as identified in other sectors (López-i-Gelats and Tábara, 2010).

Diversification, mobility, intensification, agroecology and cooperation were the main groups of adaptation strategies identified. While diversification, cooperation, mobility, and intensification are adaptation strategies widely adopted across beekeeping (Velardi et al. 2021) and farming sectors (e.g., López-i-Gelats et al. 2011, 2016), the diversity of agroecological adaptation strategies, which combine traditional and new practices, should be particularly highlighted (Fig. 4). The results indicate that the size of the beekeeping operation has a greater impact on adaptation capacity than on the perception of the transformations the sector faces. The resources available and the goals and values of professional and nonprofessional beekeepers appear to drive the implementation of different adaptation strategies. While small and medium nonprofessional operations tend to focus more on agroecological adaptation strategies (e.g., direct sale, bee-friendly management, autochthonous breeds, organic production, or reducing beehive extraction to better guarantee the health of the colony) and mutual support and collaboration, professional operations tend to focus more on diversification and mobility strategies to ensure the food security of their large apiaries (Fig. 4). Mobility provides access to more food for honeybees, although transporting colonies, especially over long distances, can negatively impact bee health (Henry et al. 2012). Although our study focuses on a specific case study region and may contain biases, our findings align with those of Velardi et al. (2021), who showed that small beekeepers and those not seeking to expand in size tend to emphasize place-based beekeeping, which is tied to landscape health in terms of forage abundance, chemical absence, and social ties to the community. The existence of different responses is significant, considering recent findings that suggest beekeeper background and practices are major drivers of honeybee colony decline (Jacques et al. 2017). Nonprofessional operations focus on improving the quality of hive products to meet the increasing demand for high-quality goods by advocating for more training, greater facilities for local sales, and more precise labeling for quality and origin (Fig. 5).

Despite the significant challenges that honeybees and beekeeping face, beekeepers also identified several emerging options for the sector’s future: (i) recognizing the ecosystem services provided by honeybees and beekeeping, either to improve the sector’s image or as a part of an economic strategy involving payment for environmental services, as noted by Potts et al. (2016) and Hung et al. (2018); (ii) the potential for agroecological management in nonprofessional beekeeping, emphasizing both the production of high-quality products and healthier products, and the use of prophylactic management options, in contrast to the current predominant therapeutic approach (in line with the IPES-Food 2016); and (iii) the recognition of beekeeping as a high-reliability economic activity, as evidenced by its role in buffering against economic crises in several regions (Bradbear 2009; Pocol and McDonough 2015; FAO 2016; Amulen et al. 2019). Finally, a major challenge remains for beekeepers to minimize the potential negative effects of high honeybee concentrations on wild bee populations and their ability to provide pollination services (Geslin et al. 2017; Alaux et al. 2019).

## Conclusions

There is wide consensus that the capacity of honeybees and beekeepers to provide their relevant contributions to society, in terms of economic return, cultural identity, and ecosystem services, depends on their ability to face the challenges posed by climate and non-climate transformations. While the literature tends to focus on the biophysical and biological implications of these transformations and trends, our study highlights the existence of a significant gap based on beekeepers’ perceptions, namely, the transformations occurring within the socioeconomic and policy domains. These included the impact of inappropriate policies, the growing recognition of societal trends that have major consequences for the sector (such as increasing demand for local, organic, and healthier products), and certain economic transformations (such as the presence of uninformative labels and the coexistence with poor-quality honey in the marketplace). Importantly, these trends may differ across countries and regions, as illustrated by the fact that dissatisfaction with public policies tends to be greater in Southern Europe.

The resources and goals available to beekeepers significantly influence the implementation of different adaptation strategies. While small and medium beekeepers tend to adopt adaptation strategies focused on agroecological management, emphasizing quality, care, and community building, large beekeepers are more likely to implement adaptation strategies centered on diversification and mobility to secure enough food for their large apiaries and production. Policy-makers, therefore, need to consider the diversity of management practices and consider the effect of farm size on the adoption of adaptation strategies. Furthermore, from this study, there are several possibilities for the sector that should be considered in policy recommendations: (i) the accurate valuation of the ecosystem services provided by beekeeping; (ii) the potential of agroecological management to deliver both high-quality and healthier products; (iii) the recognition of beekeeping as a highly reliable economic activity in changing times; and (iv) the need to promote the active participation of beekeepers in the development of agricultural and environmental policies, given their close connection with nature.

## Supplementary Information

Below is the link to the electronic supplementary material.

Supplementary file1 (PDF 205 kb)

## Data Availability

Data will be made available upon reasonable request.
