# Supplementary material for: Effect of farm size on vulnerability in beekeeping: Insights from mediterranean Spain
Source: Ambio. 2024 Dec 11;54(4):696–713. doi: 10.1007/s13280-024-02099-0 (PMC11871193; doi:10.1007/s13280-024-02099-0)
Supplement: Supplementary file 1 — Supplementary file1 (PDF 205 kb) [file 13280_2024_2099_MOESM1_ESM.pdf]

Supplementary information

This supplementary information has not been peer reviewed.

Title: **Effect of farm size in the vulnerability of beekeeping: insights from Mediterranean Spain**

Structured interview

Part I: Introduction

Family tradition? ___Yes ___ No. Age? ___
Full-time dedication___; part-time___.

- If part-time, what other activity do you combine it with? ________________________________
____

Number of workers? _____; Family members___, Hired____.
Number of beehives? ___. Recent evolution? ___increase; ___stable; ___ decrease.
What products do you produce? ___ honey; ___ pollen; ___ propolis; ___ wax; ___royal jelly; ____greenhouse; ___ others. Which ones? ____

Type of commercialization (%): ___ self-consumption; ___ direct sale; ___ intermediary: ___others. Which ones? ____

Why do you engage in beekeeping? ____
____
Would you advise your children/young people to engage in it? ___ Yes; ___ No.
Why? ____

Part II: Changes and Transformations

What climatic changes have you observed? Effects on your activity?

Changes	Mark those detected	Observations
Shift in seasons		
Increase in maximum Temperature (especially in summer; less in winter)		
Decrease in minimum Temperature (greater thermal fluctuation)		
Droughts (less rain in summer and spring)		
Lower relative humidity/less cloudiness		
Extreme events (floods, winds…)		
Increase in wind speed		
Decrease in river flow		
Others…		

What political trends are affecting your activity?

Changes	Mark those detected	Observations
Agro-environmental schemes (CAP) (pollination of natural areas)		
Promotion of organic production		
Pollination premium		
Local product labelling		
Assistance for coexisting with bee-eaters (*Merops apiaster*)		
Marginalization of beekeeping (e.g., *pinyolà*)		
Hyper-sanitary regulations		
Subsidy hunting (e.g., maintaining colonies in a deficient state for subsidy collection)		
Outdated health programs		
Others…		

What economic trends are affecting your activity?

Changes	Mark those detected	Observations
Displacement of settlements due to conflict with other uses		
Increase in fuel price		
Pesticide abuse (e.g., neonicotinoid insecticides)		
Monocultures nearby		
GMOs nearby		
Labelling fraud		
Honey adulteration		
Honey import and mixing		
Over-exploitation (more colonies and more settlements nearby)		
Others…		

What social trends are affecting your activity?

Changes	Mark those detected	Observations
Greater awareness of the value of bees		
Increase in training activities		
Incorporation of more beekeepers, but with little tradition		
Increasing demand for healthy products (products derived from the beehive)		
Increase in ecological sensitivity in producers		
Increase in demand for organic products		
Increase in appreciation of local products		
Theft of bee colonies		
Others…		

What ecological trends are affecting your activity?

Changes	Mark those detected	Observations
Expansion of bee enemies (e.g., bee-eaters, Asian hornets, small hive beetles…)		
Emergence and continuation of pathologies and syndromes		
Loss of biodiversity and habitat loss		
Disappearance of wild honey bee hives		
Decrease in flowering period		
Concentration of flowering periods		
Hybridization of native honey bees with foreign honeybees		
Pollination crisis		
Depopulation		
Wild fires		
Introduction of exotic species		
Others…		

What technical/productive trends are affecting your activity?

Changes	Mark those detected	Observations
Abuse or misuse of veterinary treatments		
Prophylaxis gaining ground over therapeutics		
Use of foreign honey bees (more docile, but less adapted)		
Lack of training in beekeepers (- sanitary rigor)		
Abuse or misuse of veterinary treatments		
Prophylaxis gaining ground over therapeutics		
Use of foreign honey bees		
Others…		

Part III: Adaptation

What strategies are there to cope with changes and impacts for beekeepers and beekeeping?

DIVERSIFICATION STRATEGIES	Mark those detected	Observations
Economic diversification of the beekeeping family		
Diversification of sites		
Diversification in the production of hive products		
Diversification of bee management		
Specialization in less common hive products (e.g., queen breeding, swarms, hive rental for crop pollination, pollen...)		
Processed products derived from the hive (e.g., body creams…)		
Others…		

MOBILITY STRATEGIES	Mark those detected	Observations
Changes in transhumance routes		
Changes in settlements (wind shelter and sun exposure...)		
Increase in mobility distances		
Adoption of sedentary beekeeping		
Transhumance		
Others…		

INTENSIFICATION STRATEGIES	Mark those detected	Observations
Increased use of inputs (e.g., systematic or planned health treatments...)		
Use of artificial feeding for winter support		
Wholesaling		
Purchase of lots of new honey bees		
Purchase of honey bees from foreign breeds, more docile		
Others…		

AGROECOLOGY STRATEGIES	Mark those detected	Observations
Organic production (certified or not)		
Use of the native black bee		
Low hive densities (e.g., less than 50 hives)		
Low apiary densities (e.g., less than 5 km apart)		
Adaptation of management to the nature of the bee (e.g., vertical hives)		
Proximity sales (direct sales)		
Quality product brand		
Local product brand		
Consumer education		
Measures to ensure animal welfare		
Hive location (e.g., Hartmann lines)		
Others…		

COOPERATION AND MUTUAL SUPPORT STRATEGIES	Mark those detected	Observations
Family labour		
Labour exchange		
Payment in kind for services tobeekeeper (e.g., honey for settlement)		
Participatory health (beekeeper awareness to compensate for lack of technicians)		
Assembly cooperativism in beekeepers' organization		
Others…		

AGROECOLOGY STRATEGIES	Mark those detected	Observations
Adaptation of management to sanitary treatment (e.g., preventive management, application when fewer broods...)		
Change of genetic line (e.g., changing queen)		
Prioritization of replacement over productivity/profitability of the apiary (e.g., colony division in spring)		
Abandonment of beekeeping activity		
Alternation of treatments for the same pathology (e.g., to avoid generating tolerance)		
Training		
Research		
Others…		

Part IV: Needs and Potentialities

What needs do you identify in the sector? ____
____
____

What potentialities? ____
____
____
